# Fast-spreading SARS-CoV-2 variants: challenges to and new design strategies of COVID-19 vaccines

**DOI:** 10.1038/s41392-021-00644-x

**Published:** 2021-06-09

**Authors:** Weilin Zhou, Wei Wang

**Affiliations:** State Key Laboratory of Biotherapy and Cancer Center, West China Hospital, Sichuan University, and Collaborative Innovation Center for Biotherapy, Chengdu, PR China

**Keywords:** Infectious diseases, Infectious diseases

The COVID-19 pandemic caused by severe acute respiratory syndrome coronavirus 2 (SARS-CoV-2) is still threatening global health. According to the latest data, the number of diagnosed cases has exceeded 100 million. Comfortingly, experiences have been accumulated in preventing and treating COVID-19 through virological, immunological, epidemiological, and clinical investigations of this disease.^1^ Besides, the continuous advancement of different vaccines brings the dawn to defeat the epidemic.^2^ However, the emergence of fast-spreading SARS-CoV-2 mutant strains (B.1.1.7, B.1.351, and B.1.1.28.1) was reported at the end of 2020, causing concern to prevention and treatment of COVID-19. It is speculated that the emergence of the SARS-CoV-2 variants may portend a new phase of the pandemic.^3^

## Emergence and global spread of SARS-CoV-2 variants

The SARS-CoV-2 is a kind of RNA virus. Due to the lack of a mismatch repair mechanism, the virus replication process is accompanied by a high mutation rate.^4^ Hence, the mutations of the coronavirus are commonsensical and predictable. Mutations could make the virus more contagious and difficult to be eliminated. For instance, the D614G (the amino acid at position 614 was mutated from aspartic acid to glycine) variant, identified by Korber et al.^5^, is more transmissible and had been dominated worldwide.^6,7^ At present, three novel variants, B.1.1.7, B.1.351, and B.1.1.28.1, have rapidly spread worldwide, causing concerns about the prevention and treatment of COVID-19.

**Fig. 1:**
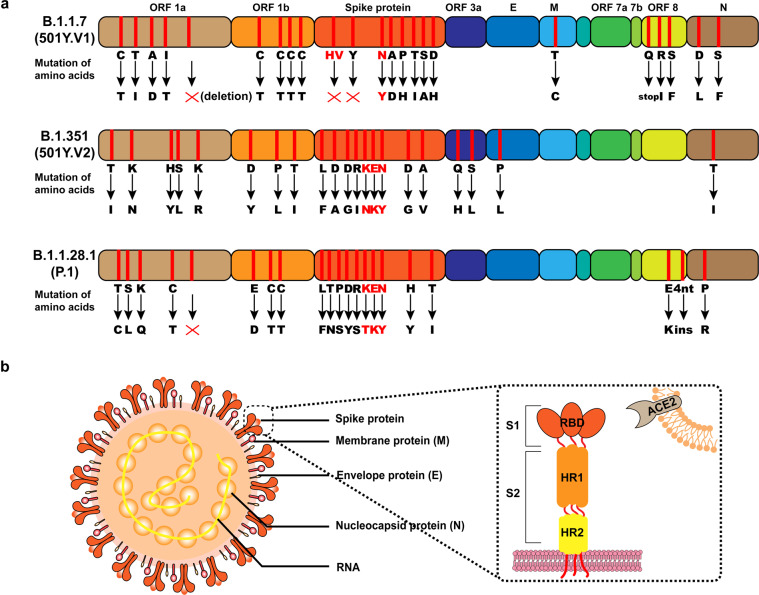
The information of fast-spreading SARS-CoV-2 variants. **a** Detailed amino acid mutations of SARS-CoV-2 variants (B.1.1.7, B.1.351, B.1.1.28.1) and key mutations are marked in red. **b** The major structure of SARS-CoV-2, including spike protein, membrane protein, envelope protein, nucleocapsid protein and RNA. As a transmembrane protein, angiotensin-converting enzyme 2 (ACE2) serves as the main entry point into cells for SARS-CoV-2

The B.1.1.7 (known as 20I/501Y.V1 or VOC 202012/01) was firstly isolated and identified in Kent and Greater London, the United Kingdom. Within several weeks, the new strain swept across the UK and was detected in numerous countries. The variant emerged with multiple mutation sites, including six synonymous mutations, 13 non-synonymous mutations, and four deletion mutations (Fig. 1a).^8^

The mutant strain B.1.351, also known as 20H/501Y.V2, was first reported by the Centre for the AIDS Programme of Research in South Africa on December 18, 2020. The lineage was early detected in the coastal areas of the Eastern Cape of South African and predominated the Eastern Cape and Western Cape within a few weeks.^9,10^ The new lineage emerged with 21 mutations,^10^ among which nine mutations were identified in the spike protein region (Fig. 1a).

The lineage B.1.1.28.1 (also known as P.1) was first reported by the National Institute of Infectious Diseases in Japan on January 6, 2021, in four travelers from Brazil. The P.1 variant outbreak had mainly found in Manaus,^11,12^ which had been experienced widespread infections in May, 2020. This strain carries 21 mutations, including one insertion, one deletion, four synonymous mutations, and 15 non-synonymous mutations (Fig. 1a). Compared with 501Y.V1 and 501Y.V2, the P.1 variant emerged with more changes (10 mutations) in spike protein.^12^ A recent study pointed out the P.1 variant caused the second outbreak in Manaus,^11^ raising the concern that immune evasion is triggered by new lineages.

Furthermore, several new SARS-CoV-2 variants have been isolated in many states of the US recently. The team at Southern Illinois University had been identified a new variant in the United States. The variant named 20C-US can be traced in May of 2020, and the main mutation occurs in Q677 and Q173 of the spike protein.^13^ The Q677H mutation near the protease cleavage site may affect the stability of the spike protein. The detailed biological effects and virus characteristics of 20C-US remain to be characterized. Researchers have reported a novel variant, called CAL.20C, appearing in Southern California. The strain was derived from cluster 20C and had five unique mutations (one in ORF1a: I4205V, one in ORF1b: D1183Y, three in spike protein: S13I, W152C, and L452R).^14^ The novel strain spans the B.1.427 and B.1.429 lineages, accounted for more than 50 percent of Los Angeles sequence samples. Scholars speculate that the CAL.20C may be one reason for the recent surge in California cases. The new SARS-CoV-2 variant, known as B.1.526, has been identified by the Columbia University. This newly identified strain emerges with multiple mutations in the spike protein, including L5F, T95I, D253G, E484K, D614G, and A701V.^15,16^ The new strain is spreading rapidly, and the number of patients infected with the variant has accounted for more than 20% of New York cases. Besides, the variant named B.1.525 has also spread in New York City. The B.1.525 lineage was firstly identified in the UK on December 15, 2020 and became the dominant lineage in Nigeria. It has four mutations (Q52R, E484K, Q677, and F888L) in the spike protein region and a deletion mutation (ΔH69/ΔV70) similar to B.1.1.7 lineage. According to the latest World Health Organization (WHO) data, we summarized multiple variants in Table 1.

**Table 1:** Overview of information on SARS-CoV-2 variants

Next strain clade	PANGOLIN Lineage	Alternate name	First detected by	Date	Key mutations in spike protein	Pathogenicity^a^
20I/501Y.V1	B.1.1.7	VOC 202012/01	United Kingdom	September 2020	H69/V70 deletion; Y144 deletion; N501Y; A570D; and P681H	Transmissibility increased (36–75%);^51^ Slight reduction in neutralization capacity^52^
20H/501Y.V2	B.1.351	VOC 202012/02	South Africa	August 2020	L242/A243/L244 deletion; K417N E484K, N501Y	More transmissible than previously circulating variant;^53,54^ reduction in neutralization capacity^43,55^
20J/501Y.V3	B.1.1.28.1	P.1	Brazil/Japan	December 2020	K417T, E484K; N501Y	More transmissible than previously circulating variant;^56^ reduction in neutralization capacity^57^
20C	B.1.525	/	United Kingdom and Nigeria	December 2020	H69-V70 deletion; Y144 deletion; Q52R; E484K; Q677H; D614G; and F888L	Under investigation
20C/S.452R	B.1.427/B.1. 429	CAL.20C/L45 2R	the United States	June 2020	L452R; W152C; S13I; and D614G	Under investigation
20B/S.484K	B.1.1.28.2	P.2	Brazil	April 2020	L18F; T20N; P26S; F157L; E484K; D614G; S929I; and V1176F	Under investigation
/	B.1.1.28.3	P.3	Philippines and Japan	February 2021	141–143 deletion; E484K; N501Y; and P681H	Under investigation
20C	B.1.526 (with E484K or S477N)	/	the United States	November 2020	L5F; T95I; D253G; D614G; A701V; and E484K or S477N	Under investigation
20C	B.1 descendant with 9 mutations	/	France	January 2021	G142 deletion; D66H; Y144V; D215G; V483A; D614G; H655Y; G669S; Q949R; and N1187D	Under investigation

^a^Descriptions of variants’ pathogenicity are subject to ongoing investigation and continuous revision

## Key mutations in the variants affect the biological function of SARS-CoV-2

Recent studies found that only the variants bearing mutations with significant biological functions exhibited high transmissibility, suggesting that these key mutations may affect the severity of COVID-19, viral spreading and escape of natural or vaccine-induced immunity.

The SARS-CoV-2 infects cells of the human through the binding of angiotensin-converting enzyme 2 (ACE2) by RBD of Spike protein (Fig. 1b). It seems that these key mutations affected the binding ability to ACE2. The variants discovered in the UK, South Africa, and Brazil have a substitution at position 501 of the spike protein (N501Y), which seems to enhance the binding ability to ACE2. Andersen et al. found that six amino acid residues of RBD are critical for the binding capacity of SARS-CoV-2 to ACE2 receptors, including L455, F486, Q493, S494, N501, and Y505.^17^ Residues N501 interact with a salt bridge D38-K353 of ACE2.^18^ This function contributes to increasing the binding ability to ACE2.^19^ Qin et al. revealed that N501Y mutation potentially associated with the increased virulence in a mouse model.^18,20^ Bloom’s work also mentioned that the N501 site mutation of RBD could enhance affinity notably.^21^ These preliminary pieces of evidence indicate that the N501Y mutation may increase transmissibility.^8,22^

Besides, Kristian Andersen identified another notable feature of SARS-CoV-2 that the spike protein has a functional polybasic (Furin) cleavage site. Once the stability of spike protein declined due to cleavage by Furin proteases, it’s possible to increase the binding ability to ACE2 receptor markedly.^23^ Unfortunately, the B.1.1.7 strain emerged with P681H mutation near the protease cleavage site, threatening spike protein stability.

The E484K mutation is coincidently found in several variants, including B.1.351, B.1.1.28.1, B.1.525, and B.1.526. This mutation occurred at critical sites in the receptor-binding motif (RBM) of the RBD. As the central functional motif, RBM is relatively unconserved and directly affects the binding to the human ACE2 receptor.^24^ The E484 interacts with the hotspot residue of human ACE2. Some evidence indicated that the E484K mutation might increase the immunological resistance of variants to neutralization of several monoclonal and human serum antibodies. Whelan et al. isolated 48 escape mutants by using a chimeric virus and 19 anti-RBD monoclonal antibodies. Subsequently, they used COVID-19 vaccine-elicited sera samples to detect the escape of mutants. All four mutants undergoing substitution at E484 are resistant to neutralization of human immune serum.^25^ Bloom et al. had also observed that the mutant at E484 could significantly avoid recognition by polyclonal human serum antibodies.^26^ Based on current data, scholars speculate that the emergence of the E484K mutation seems to have changed the antigenicity of SARS-CoV-2. Therefore, immune evasion is likely to occur in the novel strain B.1.351 that bears the E484K mutation.

Residues K417 ensures the normal binding affinity of coronavirus by forming a salt bridge with D30 of hACE2. The results of deep mutational scanning indicate that K417N/T mutation seems to have minimal impact on binding ability.^21^ However, Qin et al. generated a mouse-adapted strain of SARS-CoV-2 (MASCp6), bearing both N501Y and K417N mutations, which showed 100% fatal rate to aged male mice.^27^ This result perhaps reminds us to consider the infectivity and pathogenicity of 501Y.V2 for the aged population. Some studies found that L452R mutation weakens the binding ability of convalescent patients’ antibodies and serum to spike protein.^26,28,29^ The Q677 mutation has been detected in at least seven SARS-CoV-2 variants. However, there is no sufficient evidence to prove its impact on the pathogenicity of the variants.^30^

In addition to the spike protein mutation, the Q27 stop mutation in the ORF8 region inactivates the ORF8 function by truncating the protein. A similar situation occurred in Singapore in March 2020. This variant named Δ382 has a deletion of 382 bp in the ORF8. It is found that Δ382 variants showed significantly higher replicative fitness in vitro, but the patients infected by this variant have no different viral load compared with that of wild type.^31^ The emergence of multiple SARS-CoV-2 strains with ORF8 deletion worldwide indicates that ORF8 inactivation may be associated with the adaptive evolution of SARS-CoV-2. Outside of non-synonymous mutation, the HV 69-70 deletion has been detected in multiple lineages. It seems to facilitate the escape of the coronavirus from the host’s immunological response. For example, the variant N439K contained HV 69-70 deletion showed partial immune evasion, the variant Y453F found in mink increased the binding ability to ACE2.^24^

Although the evidence of antigen drift for SARS-CoV-2 is still insufficient, it is conceivable that the virus can acquire immunological resistance or other characteristics due to the accumulation of mutations. Coincidently, these mutations of new variants showed similar substitutions at the mutational sites. However, the emergence locations of the variants are geographically distant, indicating that the underlying mechanism by which the mutation is driven may share some similarities. It is, therefore, worthy of unveiling the biological function result from these mutations for the prevention and treatment of COVID-19.

## Current status of the COVID-19 vaccine development

Under the pressure of the COVID-19 pandemic, the speed of vaccine development and application is unprecedented. These vaccines can be divided into four categories: inactivated virus vaccine, nucleic acid vaccine, protein subunit vaccine and adenoviral vector-based vaccine.^32^ More than 70 preclinical vaccines have been tested in animals, and 86 candidates have entered the clinical trials phase. But only 13 vaccines (Table 2) have either been approved for clinical application or released data from phase III clinical trials.^2^ To date, more than 700 million doses of vaccination have been initiated in 115 countries worldwide, among which China and the United States vaccinated more than 100 million doses.

**Table 2:** SARS-CoV-2 Vaccines in phase III clinical trials and their antigen targets

Vaccine categories	Vaccine name	Antigen	Developer	Clinical Phase	Identifier
Inactivated virus vaccine	BBIBP-CorV	The whole virus	Beijing Institute of Biological Products, Sinopharm, and Institute of Viral Disease Control and Prevention	Phase III	ChiCTR2000034780, NCT04560881
	CoronaVac (formerly PiCoVacc)	The whole virus	Sinovac Biotech and National Institute for Communicable Disease Control and Prevention	Phase III	NCT04456595, 669/ UN6.KEP/EC/2020, NCT04582344, NCT04617483
	None	The whole virus	Wuhan Institute of Biological Products, Sinopharm, and Wuhan Institute of Virology, Chinese Academy of Sciences	Phase III	ChiCTR2000034780, ChiCTR2000039000, NCT04612972
	Covaxin (also known as BBV152A,B,C)	The whole virus	The Indian Council of Medical Research and the National Institute of Virology	Phase III	CTRI/2020/11/028976
Protein subunit vaccine	EpiVacCorona	chemically synthesized peptide antigens of SARS-CoV-2 proteins	Vector Institute	Phase III	NCT04527575
	NVX-CoV2372	Full- length S with two proline substitutions and three mutations at the furin cleavage site	Novavax	Phase III	2020-004123-16, NCT04611802
	ZF2001	RBD-dimer	Anhui Zhifei Longcom and the Chinese Academy of Medical Sciences	Phase III	ChiCTR2000040153
Adenoviral vector-based vaccine	Sputnik V (also known as Gam-Covid-Vac)	Full-length Spike protein	The Gamaleya Research Institute	Phase III	NCT04530396, NCT04564716, NCT04642339
	AZD1222 (also known as ChAdOx1)	Full-length Spike protein	The University of Oxford and AstraZeneca	Phase III	ISRCTN89951424, NCT04516746, NCT04540393, CTRI/2020/08/ 027170
	Convidecia (also known as Ad5-nCoV)	Full-length Spike protein	CanSina Biological Inc. and Institute of Biology at the country’s Academy of Military Medical Sciences	Phase III	NCT04526990, NCT04540419
	Ad26.COV2.S		Johnson & Johnson and Beth Israel Deaconess Medical Center	Phase III	NCT04614948, NCT04505722
mRNA vaccine	Comirnaty (also known as tozinameran or BNT162b2)	Full-length Spike protein with two proline substitutions	Pfizer and BioNTech	Phase III	NCT04368728
	mRNA-1273	Full-length Spike protein with two proline substitutions	Moderna	Phase III	NCT04470427

As the worldwide application of the COVID-19 vaccine, the side effects of vaccination have arisen the concern of society. According to the Centers for Disease Control and Prevention (CDC) and WHO, common adverse events after vaccination include headache, injection site pain, fatigue, dizziness, nausea, chills, pyrexia, etc.^33^ In the US, ~372 cases per million doses of mRNA vaccines (BNT162b2 or mRNA-1273) had been reported with non-serious adverse reactions. According to the UK safety-monitoring system, there are about 4000 adverse reactions per million doses of the ChAdOx1 vaccine (AZD1222). The Phase I/II clinical trial data of inactivated virus vaccine, including CoronaVac and two inactivated virus vaccines developed by the Sinopharm, showed that most of the adverse events were common side effects and none were serious.^33–35^ To date, no death case has been reported directly attributable to the vaccination.

In brief, there is no doubt that the current vaccines are safe. However, concerns about the effectiveness of vaccines have also arisen with the emergence of variants. We still need more clinical data to monitor the effects of vaccines for a long time.

## The impacts of SARS-CoV-2 variants on the protective efficacy of COVID-19 vaccines

As a crucial strategy to combat the COVID-19 pandemic, vaccination is carried out globally. However, with the emergence of multiple SARS-CoV-2 variants, whether the vaccines’ effectiveness will be impacted has become the core issue of the global discussion. Present investigations demonstrated that SARS-CoV-2 variants substantially affect the efficacy of COVID-19.

The mRNA vaccine from Pfizer was the first approved COVID-19 vaccine. Shi et al. assessed the neutralization of BNT162b2 vaccine-elicited sera by using engineered mutant viruses. The three engineered variants, including N501Y variant, 69/70-deletion + N501Y + D614G variant and E484K + N501Y + D614G variant, showed minimal effect on neutralization of twenty BNT162b2 vaccine-elicited sera.^36,37^ Moreover, Nussenzweig et al. investigated the antibody and memory B cell responses in 20 participants who received either mRNA-1273 vaccines or BNT162b2 vaccines. They found that the neutralizing activity of vaccine-elicited sera against pseudoviruses (E484K, N501Y, and K417N-E484K-N501Y combination) was reduced.^38^ Another research also demonstrated that E484K mutant strain significantly reduced the neutralizing activity of human convalescent and post-vaccination sera.^39^ Researchers used convalescent sera, vaccine-elicited sera (mRNA-1272 and NVX-CoV2373) and monoclonal antibodies to assess the neutralization phenotype of the pseudoviruses of 501Y.V1, 501Y.V2 and P.1. They observed a decrease in neutralizing activity.^7,40,41^ However, the significant limitation of the current studies is that the engineered pseudovirus cannot fully present the biological properties of the authentic viruses.

In February 2021, an investigation reported that two approved vaccines (BBIBP-CorV and ZF2001) still have the protective efficacy to 501Y. V2 authentic virus, although neutralization titer of post-vaccination sera against 501Y.V2 declined 1.6-fold. These data indicated that the 501Y.V2 showed more resistance to the vaccinee serum.^42^ Sigal et al. also have found that plasma from convalescent patients infected with no-CoV variant (the variants usually showed the D614G mutation) have reduced neutralizing ability to 501Y.V2 variant, but plasma from convalescent patients infected with 501Y.V2 only showed moderate reduction of neutralizing to the no-COV variant.^43^

Recently, Wang et al. assessed the immunological resistance of the variants to neutralization by using convalescent sera and sera from participants received inactivated-virus vaccines (BBIBP-CorV or CoronaVac). Their findings indicated that the neutralization of convalescent or BBIBP-CorV-elicited sera against B.1.1.7 variant reduced slightly, whereas the neutralization against B.1.351 reduced significantly.^44^ The two variants showed more resistance to the CoronaVac-elicited serum than the wild-type virus. Several experiments have also been exerted to investigate the immunological resistance of variants to the neutralization of antibodies or sera.^45–48^

The biotech firm Novavax recently disclosed the results of phase III clinical trials of NVX-CoV2373 for variants. The protective efficacy to 501Y. V1 (B.1.1.7) and 501Y. V2 (B.1.351) is apparently different. The effectiveness against 501Y.V1 is more than 85% and the efficacy against 501Y.V2 is less than 50%.^49^ This finding indicated that SARS-CoV-2 variants also challenge recombinant protein vaccine.

In general, the available data have indicated that the variant of SARS-CoV-2 may have the ability to resist vaccine-induced immunity. These studies suggest that we should try to update the therapeutic strategy and vaccine design against the challenges from variants.

## Design strategies of COVID-19 vaccine against challenges from the SARS-CoV-2 variants

At present, most variants emerged locally and did not spread to other regions. Even if the variants partially escape the neutralization of antibodies elicited by the vaccination, theoretically it still cannot completely resist to the recognition of the existed antibodies since the variants share similarity of the antigenicity with the original virus. Therefore, organized and extensive vaccination by currently available vaccines is still necessary.

To fight against the challenges of SARS-CoV-2 variants, the development of vaccines effective to neutralize the variants is urgent.^50^

The spike protein of SARS-CoV-2 is the most prevailing target for COVID-19 vaccine development. The emergence of variants with mutations in spike protein may disrupt some original vaccine development schedules. Although the spike protein structure of the variant might be changed, the designs of vaccines for the variants always target the spike protein. For the development of nucleic acid vaccine, protein subunit vaccine, and adenoviral vector-based vaccine, it is relatively easy to update the vaccine antigen the same with that of the variant. In principle, the vaccine can be updated only by modifying the gene sequence of the spike protein. However, the consequence of modification is to be investigated, especially for the safety, efficacy of the original virus and variants. Many institutions and pharmaceutical companies are currently focusing on the development of new vaccines for the SARS-CoV-2 variants. It is worth noting that three angles are critical for the design of the new vaccines. (i) design new vaccines against variants and vaccinate individuals based on initial vaccines to obtain fresh immunological memory (ii) Try to develop “multivalent vaccine” to gain immunity to multiple variants. (iii) obtain higher antibody titers by re-vaccinating the original vaccine. These investigations require a large amount of laboratory and clinical endeavors. Meanwhile, we need to closely monitor the genomic information of the virus to detect the mostly new variants. Other limitations should be broken, including insufficient vaccine manufacturing, transportation and preservation, no general guiding regulations, etc.

Coping with the life after COVID-19, we highlight several perspectives: firstly, to upgrade and develop vaccines promptly, we should continue to track the COVID-19 and detect the emergence of new variants. Secondly, no vaccine can be applied to all situations or cases. Therefore, diversified vaccine development and application are critical. Finally, we should break the barriers and promote global cooperation in research on the COVID-19. We need to share the data promptly to address the challenges of the future.
